# Aspartate β-hydroxylase as a target for cancer therapy

**DOI:** 10.1186/s13046-020-01669-w

**Published:** 2020-08-18

**Authors:** Madiha Kanwal, Michal Smahel, Mark Olsen, Jana Smahelova, Ruth Tachezy

**Affiliations:** 1 Department of Genetics and Microbiology, Faculty of Science, Charles University, BIOCEV, Vestec, Czech Republic; 2 Department of Pharmaceutical Sciences, College of Pharmacy – Glendale, Midwestern University, Glendale, AZ USA; 3 Crenae Therapeutics, Phoenix, AZ USA

**Keywords:** ASPH, Small molecule inhibitor, Metastasis, Immunotherapy

## Abstract

As metastasis is a major cause of death in cancer patients, new anti-metastatic strategies are needed to improve cancer therapy outcomes. Numerous pathways have been shown to contribute to migration and invasion of malignant tumors. Aspartate β-hydroxylase (ASPH) is a key player in the malignant transformation of solid tumors by enhancing cell proliferation, migration, and invasion. ASPH also promotes tumor growth by stimulation of angiogenesis and immunosuppression. These effects are mainly achieved via the activation of Notch and SRC signaling pathways. ASPH expression is upregulated by growth factors and hypoxia in different human tumors and its inactivation may have broad clinical impact. Therefore, small molecule inhibitors of ASPH enzymatic activity have been developed and their anti-metastatic effect confirmed in preclinical mouse models. ASPH can also be targeted by monoclonal antibodies and has also been used as a tumor-associated antigen to induce both cluster of differentiation (CD) 8^+^ and CD4^+^ T cells in mice. The PAN-301-1 vaccine against ASPH has already been tested in a phase 1 clinical trial in patients with prostate cancer. In summary, ASPH is a promising target for anti-tumor and anti-metastatic therapy based on inactivation of catalytic activity and/or immunotherapy.

## Background

Cancer is a multifactorial disease with an approximate 9.6 million fatalities in 2018. Worldwide, it is the second leading cause of death [1]. The complex modifications in the genome affected by the interactions between host and environment lead to cancer development and progression. Despite advancements in characterizing the molecular mechanisms of oncogenesis, tumor progression and metastasis [2], delayed cancer detection, limited surgical options, therapeutic resistance, and tumor recurrence are serious obstacles in decreasing the prevalence and fatality rate of cancer. Since metastasis is the primary cause of deaths from cancer, the design of therapeutic approaches that target mechanisms of tumor-cell migration and invasiveness is essential. In this regard, a growing number of investigations of signaling pathways involving products of oncogenes and tumor suppressor genes in human carcinomas has helped to elucidate the mechanisms underlying malignant transformation of cells and facilitated the development of new and more efficient therapeutic methods.

Aspartate β-hydroxylase (ASPH) has been identified as one of the cell surface proteins associated with malignant transformation of tumor cells [3, 4]. ASPH belongs among the most important biological targets to control migration and invasion of tumor cells, as its overexpression has been observed in 70–90% of human solid tumors [5–7]. The overexpressed ASPH is transported from the endoplasmic reticulum to the plasma membrane which results in exposure of the C-terminal region to the extracellular environment where it is accessible to antibody binding. Recently, molecular targeted therapy has been developed against this target using small molecule inhibitors (SMI) that can inhibit the catalytic site in the C-terminal region. Moreover, as antigenic epitopes that reside on the ASPH protein can efficiently stimulate cluster of differentiation (CD) 4^+^ and CD8^+^ T-cell responses unique to tumor cells harboring ASPH, this enzyme can be used as a tumor associated antigen (TAA) in immunotherapy [8, 9].

## Structure of the ASPH gene and isoforms

**Fig. 1:**
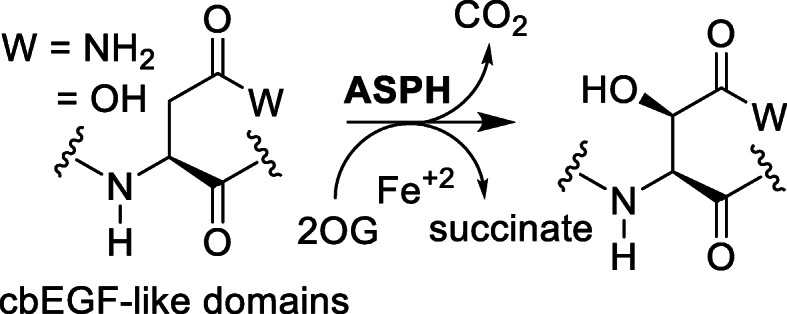
ASPH catalytic reaction. Aspartyl and asparaginyl residues in cbEGF-like domains are hydroxylated

ASPH is a type II transmembrane protein of approximately 86 kDa that belongs to the family of α-ketoglutarate-dependent dioxygenases. The β-hydroxylated products of ASPH hydroxylation were first detected in blood coagulation proteins [10–18]. ASPH was initially identified in the bovine liver as an enzyme responsible for catalyzing the hydroxylation of aspartyl and asparaginyl residues in calcium binding epidermal growth factor (cbEGF)-like domains of various proteins [19] (Fig. 1). Thereafter, the human ASPH gene was cloned and characterized [20]. This gene spanning 214,085 base pairs long region of genomic DNA and containing 33 exons is located at the position q12.3 of the human chromosome 8. The ASPH sequence is highly conserved in mammalian evolution. The sequence of the human protein is from about 85% identical to the sequences of rat and mouse analogs and the catalytic site is quite conserved among proteins of these three species [7]. The whole ASPH protein consists of five domains: an N-terminal cytoplasmic, a universal transmembrane, a positively charged luminal, a calcium binding, and a C-terminal catalytic domain [21]. Tissue specific transcription is directed from two putative promoters, P1 and P2, which differ in their regulation sequences [21, 22]. While the transcription from the P1 promoter was observed in most human tissues, the P2 promoter is activated by the calcium-dependent transcription factor myocyte enhancer factor 2 (MEF2), particularly in muscle tissues [21]. The *ASPH* gene undergoes extensive alternative splicing resulting in four protein isoforms, i.e. ASPH, humbug, junctate, and junctin [23, 24]. These proteins vary in the C-terminal region, which affects their function [25, 26]. The two longest ASPH transcript variants, that are transcribed from the P1 and P2 promoters and differ in the length of the 5′-untranslated region, encode the full-length ASPH protein. This protein contains the catalytic C-terminal domain that catalyzes the post-translational hydroxylation in the cbEGF-like domains of numerous proteins (Supplementary Fig. 1), including receptors, receptor ligands, and extracellular adhesion molecules, that influence cell motility and invasiveness [5, 25]. The truncated isoforms, humbug, junctate, and junctin, share the N-terminal part with the ASPH protein but lack catalytic function. They are involved in calcium homeostasis [27]. Humbug has a potential role in cell adhesion and calcium flux and similar to ASPH, its overexpression has been correlated with aggressive tumor-cell behavior [28]. Junctate is a sarco(endo)plasmic reticulum membrane-bound protein that is known for its function in the regulation of the intracellular Ca^2+^ concentration. Junctin is a structural membrane protein and as an integral part of the complex consisting of the ryanodine receptor, calsequestrin and triadin influences calcium release from the sarcoplasmic reticulum [24, 27, 29].

## Localization in cells, tissue distribution, and expression regulation

ASPH is predominantly a cell-surface protein [30] that is also localized in the endoplasmic and sarcoplasmic reticulum [31]. Furthermore, a recent study identified mitochondrial localization of ASPH in hepatocellular carcinoma (HCC). In that study, ASPH overexpression correlated with an instability of mitochondrial DNA and mitochondrial dysfunction that may lead to more aggressive pathological outcomes in HCC [32].

ASPH is abundantly expressed in proliferating placental trophoblastic cells [3, 33] and in decidua and endometrial glands [33] and has a potential role in placental implantation and fetal growth [34]. On the contrary, the ASPH expression in normal adult tissues is relatively low or negligible. However, ASPH expression is inappropriately activated during oncogenesis when ASPH is required for generation of malignant and metastatic phenotypes. The elevated expression of ASPH at both transcription and translation levels has been shown in a wide range of transformed cell lines as well as human carcinoma tissues including hepatocellular, pancreatic, colon, prostate, lung, breast, ovarian, and cervical carcinoma, cholangiocarcinoma, neuroblastoma, and gastric cancer (Table 1). The first study that demonstrated the significantly higher expression of both ASPH mRNA and protein in HCC and cholangiocarcinoma, relative to their normal adjacent tissue counterparts, was by Lavaissiere et al. [3]. Subsequently, they verified the role of upregulated ASPH protein production and its enzymatic function in the malignant transformation on biliary epithelium, the NIH-3 T3 cell line, and animal models [4]. The level of ASPH also correlated with cell motility and invasiveness in in vitro experiments [30, 38, 44]. In the study by Maeda et al. [36], the overexpression of the ASPH protein was in accordance with worse clinical and histopathological characteristics of the intrahepatic cholangiocarcinomas and prognosis of patients. Similar findings were obtained in other studies for hepatocellular [40, 45], non-small cell lung [46], and colon carcinomas [47] and glioblastoma multiforme [6]. Recently, the prognostic significance of 2-oxoglutarate-dependent oxygenase expression was demonstrated by analysis of expression profile datasets of 20,752 tumor samples and 881 non-tumor samples. ASPH has been identified as one of the genes which upregulated expression could serve for risk stratification of patients with 9 cancer types [48]. In glioblastoma, the prognostic significance of ASPH was suggested by profiling of alternative mRNA splicing [49].

**Table 1:** Summary of the studies, which have identified the elevated ASPH expression in human tumor tissues

Study	Tumor tissues	Positive cases of studied samples (n/n)	Detection method	Antibody (recognized region of ASPH protein)
Lavaissiere et al., [3]	Hepatocellular	4/10	IHC	FB-50 Ab (N-terminus)
	Cholangiocarcinoma	20/20		
	Breast	4/4		
	Colon	6/10		
Palumbo et al., [35]	Pancreatic adenocarcinoma	19/19	IHC	FB-50 Ab (N-terminus)
Sepe et al., [30]	Primitive neuroectodermal (medulloblastoma, neuroblastoma)	28/28	IHC	FB-50 Ab (N-terminus)
Maeda et al., [36]	Cholangiocarcinoma	42/50	IHC	FB-50 Ab (N-terminus)
Cantarini et al., [37]	Hepatocellular	13/15	IHC	FB-50 Ab (N-terminus) or 15C7 Ab (catalytic domain)
		13/15 (7.5-fold higher level of mRNA compared to normal tissue)	RT-qPCR	
Monte et al., [38]	Hepatocellular	8/8	IHC	FB-50 Ab (N-terminus)
		8/8 (7-fold higher level of mRNA compared to normal tissue)	RT-qPCR	
Yang et al., [39]	19 types of tumor tissues^a^	94/104	IHC	mAb G3 hybridoma
Wang et al.*,* [40]	Hepatocellular	150/233	IHC	Polyclonal
Dong et al., [41]	Pancreatic cancer	101/104	IHC	FB-50 mAb (N-terminus)
Tang et al., [32]	Hepatocellular	71/140	RT-qPCR	
Lin et al., [42]	Breast	127/141	IHC	FB-50 Ab (N-terminus)
Ogawa et al., [43]	Pancreatic ductal adenocarcinoma	162/166	IHC	FB-50 Ab (N-terminus)

^a^Liver, kidney, breast, cervical, ovarian, Fallopian tube, laryngeal, lung, thyroid, pancreatic, thymic, prostate, bladder, esophagus, gastric, gall bladder, colon, and rectum cancer and cholangiocarcinoma

**Fig. 2:**
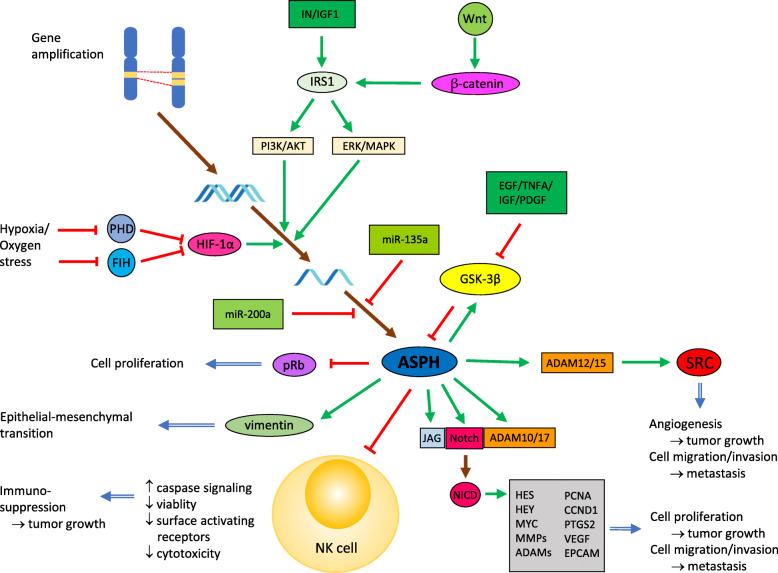
Regulation of ASPH expression and ASPH involvement in signaling pathways. The expression of the ASPH protein can be regulated at several levels. The *ASPH* gene can be amplified in tumor cells and its transcription activated by IN/IGF-1 and Wnt/ β-catenin pathways or induced by hypoxia. At the posttranscriptional level, miR-200a and miR-135a can downregulate ASPH expression. Stability of the ASPH protein can be reduced by phosphorylation with GSK-3β. Conversely*,* ASPH can enhance GSK-3β activity by inhibition of its phosphorylation with AKT and p38 kinases. ASPH also supports cell proliferation, epithelial-mesenchymal transition, migration, invasion, and angiogenesis and consequently tumor growth and metastasis by hydroxylation of the Notch receptor and ligands (ex. JAG) and interaction with pRb, vimentin and ADAMs. Finally, inactivation of NK cells by ASPH has been demonstrated. Green arrow, activation signal; red bar, inhibitory signal

ASPH gene expression is upregulated via Wnt/β-catenin [50] and insulin/insulin-like growth factor 1 (IGF1)/insulin receptor substrate 1 (IRS1) signaling [25, 37, 38] through extracellular signal-regulated kinase (ERK)/mitogen-activated protein kinase (MAPK) and phosphatidylinositol-3-kinase/protein kinase B (PI3K-Akt) pathways (Fig. 2; for review, see ref. [26]). Insulin/IGF1/IRS1 signaling affects cell growth and survival and can be involved in oncogenesis in various human tumors [51]. The β-catenin-dependent Wnt pathway regulates cell proliferation, motility, and differentiation and is one of the most frequently modified pathways in human malignancies. Upon aberrant activation of Wnt signaling, β-catenin is accumulated in the cytoplasm and subsequently translocated to the nucleus [52], where an interaction between β-catenin and T-cell factor/lymphoid enhancer-binding factor (TCF/LEF) proteins forms a transcriptional regulatory complex which enhances the expression of Wnt target genes including *IRS1* [53]. ASPH was proposed as a common link between Wnt/β-catenin and insulin/IGF1/IRS1 pathways and downstream signaling [54].

The regulation of *ASPH* gene expression in tumors might also be affected by a copy number variation. In the study by Kadota et al. [55], the *ASPH* gene locus has been identified as one of the DNA regions with focal amplification in primary breast cancer. In colorectal cancer, *ASPH* gain or amplification was found in 56% of samples [56]. Next, a suppressant role of the microRNA miR-200a in posttranscription regulation of the *ASPH* expression in hepatoma cells has been found [57]. MiR-200a belongs to miR-200 family, which plays significant role in preventing cancer initiation and metastasis (for review, see ref. [58]). Similarly, miR-135a has been shown to suppress ASPH in endometrial cancer [59].

Moreover, consistent with the protein sequence analysis that recognized numerous prospective phosphorylation sites of glycogen synthase kinase-3β (GSK-3β), casein kinase 2 (CK2), protein kinase A (PKA), and protein kinase C (PKC) on ASPH [60], several studies demonstrated that phosphorylation can regulate the ASPH protein expression [25, 52–55]. Inhibition of the GSK-3β activity did not modify mRNA expression but increased the ASPH protein level [25]. Direct phosphorylation of ASPH by GSK-3β probably decreases ASPH stability and thus reduces cell mobility [60]. ASPH protein expression was also increased by inhibitors of PKA, PKC, and CK2 [61]. Mutational analysis of potential sites of phosphorylation demonstrated complex and nonuniform effects of ASPH phosphorylation on protein expression, enzymatic activity, and subcellular localization [62, 63]. Therefore, ASPH phosphorylation probably regulates the function of this protein by various mechanisms.

ASPH expression can also be regulated by hypoxia and oxidative stress. In human neuronal cells, this effect was mediated by hypoxia inducible factor 1 alpha (HIF-1α) that is stabilized under hypoxia/oxidative stress when the prolyl hydroxylase domain (PHD) proteins and factor inhibiting HIF (FIH) are inactivated. Consequently, the HIF-1 heterodimer made up of subunits HIF-1α and HIF-1β functions as a transcription factor likely enhancing *ASPH* expression by binding to hypoxia-responsive elements [64]. In hypoxic regions of glioblastoma, both HIF-1α and ASPH were highly expressed, particularly in more aggressive mesenchymal subtype of glioblastoma, suggesting a possible involvement of ASPH in mesenchymal transition [6]. Brewitz et al. showed reduced ASPH hydroxylation activity at low oxygen concentrations and suggested an ASPH role in oxygen (hypoxia) sensing. ASPH upregulation induced by hypoxia could compensate for reduced enzymatic activity [65]. Moreover, a recent study reported an oxidative stress state of the castration-resistant prostate cancer cells upon ASPH overexpression which was reversed by silencing *ASPH* expression or generating hypoxic conditions resulting in impaired cell proliferation and invasion [66].

## ASPH protein interactions and signaling pathways

The ASPH hydroxylation consensus sequence is confined within cbEGF-like domains that are found in proteins of diverse function, including Notch receptors and ligands, clotting factors, structural proteins of the extracellular matrix, and ligands of the tyro-3/Axl family of receptor tyrosine kinases [23].

The Notch signaling cascade is a remarkably conserved pathway. Notch proteins (Notch1 - Notch4) are single-pass cell surface receptors that mediate communication between cells and their expression is crucial for proper embryonic development [67]. Notch signaling mainly results in cell differentiation but also plays a significant role in proliferation, apoptosis, and the maintenance and self-renewal of stem cells. Dysregulation of the Notch pathway is directly linked to cancer, vascular disorders, and congenital defects [68, 69]. In mammals, Notch signaling activated by binding of one of two families of canonical Notch ligands, jagged (JAG1 and JAG2) and delta like (DLL1, DLL3, and DLL4), leads to the generation of the cleaved Notch intracellular domain (NICD) fragment and its nuclear translocation. In the nucleus, the NICD fragment interacts with the DNA binding complex CSL (*C*BF-1/RBP-jκ, *S*u(H), *L*ag-1). This complex is then converted from a repressor into an activator leading to increased transcription of target genes such as hes family bHLH transcription factor 1 (*HES1*), HES with YRPW motif 1 (*HEY1*), *CD44*, epithelial cell adhesion molecule (*EPCAM*), *c-myc* proto-oncogene, matrix metallopeptidase 2/9 (*MMP2/9*), cyclin D1, cyclooxygenase 2, vascular endothelial growth factor (*VEGF*), and proliferating cell nuclear antigen (*PCNA*) [26, 41].

Upregulation of ASPH results in enzymatic modification of the cbEGF-like repeats in the Notch receptor extracellular domain and its ligands which promotes the receptor interaction with the ligands and the activation of Notch signaling [37, 41]. Furthermore, the interaction of ASPH with a disintegrin and metallopeptidase domain (ADAM) 10/17 stabilizes this complex and enhances the S2 cleavage of the Notch receptors and subsequent NICD fragment release [42]. The activation of the target genes in malignant cells increases cell proliferation, migration, and invasion [41] through the epithelial-to-mesenchymal transition (EMT) that is probably upregulated by the interaction of ASPH with vimentin [70]. Consequently, this activation supports tumor growth and metastasis. The ASPH-Notch axis also stimulates the release of exosomes that transfer proteins involved in invasion, metastasis, metabolism, and immunosuppression [42, 71].

The SRC kinase pathway is another important pathway in malignant cell transformation that regulates a complex signaling network promoting angiogenesis, invadopodia formation and maturation, and metastasis [72]. ASPH has been identified as an SRC pathway activator. Overexpressed ASPH directly interacts with ADAM12/15 and strengthens the SRC activation by these proteins which promotes MMP-mediated extracellular matrix degradation and tumor invasiveness [43].

ASPH can also contribute to malignant phenotype of cells by interaction with other proteins. Iwagami et al. revealed the interaction of ASPH with GSK-3β that prevents GSK-3β inactivation by phosphorylation with upstream kinases [73]. This mechanism was confirmed in a castration-resistant prostate cancer model [66]. GSK-3β is a multifunctional kinase that is involved in various processes including glycogen metabolism, cell division, and cell fate determination. Some types of tumors are sensitive to GSK-3β inhibitors [74]. Recently, Huang et al. elucidated a direct binding of ASPH with retinoblastoma protein (pRb) leading to pRb phosphorylation [75]. They also showed that this effect was mediated by increased binding of cyclin-dependent kinase (CDK) 2, CDK4, and cyclins D1 and E with pRb and was dependent on ASPH enzymatic activity. As phosphorylation of pRb inactivates its tumor-suppressor function, ASPH can contribute to the progression of cell cycle via interaction with pRb.

## Effect of ASPH on an immune system

Tumor generation and progression are influenced by cancer immunoediting that involves immunosurveillance and escape from a host immune system [76]. In these processes, various mechanisms of both innate and adaptive immunity are included [77]. Immune cells that infiltrate developing tumors are initially anti-tumorigenic but in tumor microenvironment, they can be modified into cells with pro-tumorigenic properties [78].

As potential targets of ASPH hydroxylation are also expressed on immune cells, this enzyme could affect the function of immune system, particularly in tumor microenvironment when ASPH is overexpressed on cancer cells. Indeed, such effect was demonstrated for human natural killer (NK) cells by using recombinant ASPH which reduced viability and cytotoxicity of these cells via enhancing caspase signaling and decreasing the surface expression of activating receptors, respectively [79]. Antibodies against ASPH inhibited these effects.

Interaction of ASPH with other immune cells has not been studied. However, we suppose possible influence of ASPH on different tumor-infiltrating cells. This assumption comes from the involvement of Notch signaling in differentiation and function of various immune cells, fibroblasts, mesenchymal cells, and endothelial cells. For instance, Notch activation contributed to stimulation of pro-inflammatory/anti-tumorigenic M1 polarization in both bone marrow-derived primary macrophages [80, 81] and tumor-associated macrophages [82]. When Notch signaling was abrogated, pro-tumorigenic M2 polarization was induced even by stimulators of M1 polarization [81]. miR-125a has been identified as a downstream mediator of Notch signaling in macrophages [82]. Similarly, the Notch pathway plays an important role in differentiation of other types of myeloid cells and probably all subsets of CD4^+^ and CD8^+^ T cells [83]. Different Notch receptors and their interaction with different ligands contribute to these processes [84]. Moreover, non-canonical Notch signaling is implicated in regulation of immune cells [85]. While activation of Notch signaling in some cells (e.g. T helper 1 cells, cytotoxic CD8^+^ T cells, and M1 macrophages) supports induction of immune reactions including anti-tumor immunity, in other cells (particularly regulatory T cells) it leads to immunosuppression [86]. Thus, immunostimulatory effect of Notch signaling is often inhibited in tumor microenvironment to enable the tumor cells to escape from the host immunity [84]. Therapeutics affecting Notch signaling in malignant diseases are being developed and tested in clinical trials but their effects on immune reactions and possible combination with immunotherapy have not been properly studied.

## ASPH as a therapeutic target

Oncogenic abilities of ASPH have been experimentally demonstrated using tumor cell lines and mouse and rat models of different types of human tumors with ASPH overexpression, including cholangiocarcinoma [4, 44, 75, 87, 88], hepatocellular carcinoma [5, 37, 38, 57, 62, 70, 73], neuroblastoma [30], pancreatic cancer [7, 41, 43, 71], glioma [6], breast carcinoma [42], castration-resistant prostate cancer [66], and colorectal cancer [56]. In studies analyzing ASPH function, various approaches were utilized to reveal signaling pathways affected by ASPH. Particularly, ASPH expression was diminished by using small interfering RNAs [32, 34, 38, 56, 64], short hairpin RNAs [6, 73, 87] or the CRISPR/Cas9 system [42, 56, 73]. The importance of ASPH enzymatic activity in these processes was shown by the site directed mutagenesis [4, 62, 63] or treatment by SMIs [5, 41, 42, 56, 71, 87]. In vitro assays showed ASPH involvement in cell proliferation, migration, and invasion. Cellular alterations included EMT, inhibition of apoptosis, and stemness acquisition. Tumor growth and invasiveness could further be supported by ASPH-induced extracellular matrix degradation, angiogenesis, and transendothelial migration. Notch and SRC signaling are probably major pathways influenced by ASPH (Fig. 2) and contributing to increased aggressiveness of tumor cells that was verified in in vivo models. Thus, these studies also demonstrated that ASPH is a suitable target for cancer treatment, especially by SMIs or immunotherapy.

### Small molecule inhibitors

**Fig. 3:**
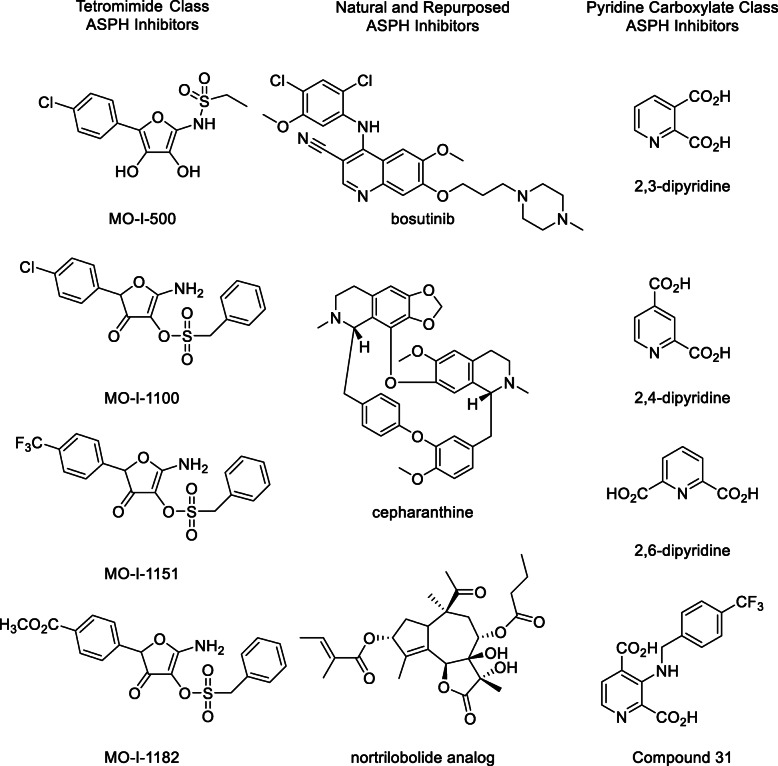
Small molecule inhibitors of ASPH

SMIs of ASPH (Fig. 3) have been developed and used to test the role of ASPH in a wide range of cancer models, including subcutaneous, orthotopic, and patient derived xenograft in vivo models [5, 42, 43, 87]. A small, orally bioavailable inhibitor has several intrinsic advantages over immunotherapy approaches. Not only can these inhibitors inhibit the catalytic activity of ASPH unlike conventional antibodies that simply bind to the protein, but they can also penetrate into the cell and inhibit ASPH catalytic activity in the endoplasmic reticulum. Different cancers have different ASPH expression patterns, and while surface expression is quite common in pancreatic cancer and hepatocellular carcinoma, intracellular overexpression patterns have also been observed [75].

The first ASPH SMIs published were the tetronimides MO-I-500 and MO-I-1100. Tetronimides were originally synthesized in 1953 by Dahn [89], and are redox-active mimics of ascorbic acid and 2-oxoglutarate. MO-I-500 is a mixed inhibitor that inhibits both ASPH and the Fat Mass and Obesity protein (FTO) [90], and is not only orally bioavailable, but also can penetrate the blood-brain barrier. MO-I-1100 is a more potent inhibitor of ASPH and is also more selective [5]. Despite investigation against a wide range of iron-dependent dioxygenases and kinases, there are no other known enzymatic targets for MO-I-1100. Enhanced activity was observed by replacing the chlorine with a trifluoromethyl group [87] as in MO-I-1151 and even a greater improvement in in vivo activity was found by replacing the trifluoromethyl group with a carboxymethyl group as in MO-I-1182, although it is not yet clear if the nature of this enhancement is due to increased inhibitory activity or enhanced solubility parameters. MO-I-1182 is reported to have the ability to suppress invasive activity at a concentration of 50 nM [43]. SMIs of ASPH have a characteristic in vitro concentration dependent profile, where the activity of the SMI plateaus at value around 50% viability [5], emphasizing the non-cytotoxic properties of this class of inhibitors.

Natural products, and inhibitors of other enzymes that have been repurposed as ASPH inhibitors have also recently been reported in the patent literature, including bosutinib (CN201910141421.9), cepharanthine (CN201910141432.7), and guaianolides related to nortrilobolide (CN201910141418.7; CN201910457588.6). Bosutinib is a well-known inhibitor of BCR-ABL and SRC tyrosine kinases approved for the treatment of chronic myelogenous leukemia [91]. Cepharanthine is a natural product sesquiterpene with complex anticancer activity, including AMP-activated protein kinase **(**AMPK) activation and nuclear factor kappa B (NFκB) inhibition [92]. Nortrilobolide and related compounds are reported to be potent cytotoxic agents with subnanomolar sarco/endoplasmic reticulum calcium ATPase (SERCA) inhibition [93]. Recently, a family of potent pyridine dicarboxylates have also been published [65] utilizing a mass spectrometry-based inhibition assay [94]. These compounds are related to known iron-dependent dioxygenase inhibitors 2,3-pyridine dicarboxylate, 2,4-pyridine dicarboxylate and 2.6-pyridine dicarboxylate. The synthesized pyridine dicarboxylates were assayed for activity against a range of other enzymes, to include PHD2, FIH, and lysine-specific demethylase 4E (KDM4E) in addition to ASPH, with varying degrees of selectivity. However, while cell-based activities have not been evaluated, the dicarboxylate nature of the compounds may be useful for cell surface ASPH inhibitors that may not have cell penetrating activity [94].

### Immunotherapy

ASPH can be used not only as a target of the inhibitors inactivating its enzymatic activity but also as a target of immune reactions leading to destruction of tumor cells and tumor growth suppression. Since ASPH is cell surface displayed on tumor cells, it represents a tumor-associated antigen that can be targeted by both cell-mediated and humoral immunity. As a target of humoral immunity, ASPH on the surface of cancer cells can be bound by antibodies that mediate antibody-dependent cellular cytotoxicity (ADCC), complement dependent cytotoxicity (CDC), or antibody-dependent cellular phagocytosis (ADCP) [95]. When the ASPH antigen is processed in tumor cells or antigen presenting cells, antigenic peptides are presented on these cells by human leukocyte antigen (HLA) class I or class II molecules and recognized by CD8^+^ or CD4^+^ T lymphocytes, respectively [96], that can be stimulated by immunization breaking tolerance to self-antigens [97].

Induction of ASPH-specific CD4^+^ and CD8^+^ T cells was examined in blood samples of HCC patients. Using synthetic peptides derived from ASPH after prediction of HLA class I- and HLA class II-restricted epitopes, it has been found that ASPH is a highly immunogenic protein that activates both types of analyzed T cells [9]. Thus, efficient anti-tumor reactions could be stimulated by immunization.

The first vaccine against ASPH was based on matured dendritic cells (DC) loaded with the ASPH protein and tested in an orthotopic rat model of intrahepatic cholangiocarcinoma [98]. This study showed that vaccination stimulated cytotoxicity against cancer cells in an in vitro assay and decreased tumor growth and metastasis. Both CD8^+^ and CD4^+^ cells contributed to an anti-tumor effect induced in a mouse model of HCC by immunization with ASPH-loaded DCs [8].

The next anti-ASPH vaccine was based on a bacteriophage lambda display system. The viral capsid protein gpD was fused with the N- or C-terminus of ASPH and immunogenicity of these nanoparticle-forming constructs was verified in two mouse tumor models [99]. The vaccine PAN-301-1 containing these constructs has already been examined in a phase 1 clinical trial in 12 patients with biochemically relapsed prostate cancer [100]. This study demonstrated safety and immunogenicity of PAN-301-1 and indicated an anti-tumor effect in terms of the reduction of prostate specific antigen (PSA) or PSA doubling time. ASPH-specific immune responses were mediated by both antibodies and T lymphocytes.

As ASPH is a type II transmembrane protein, its C-terminus carrying the enzymatic domain is exposed outside cells and can be bound by antibodies that can be used for diagnostic and therapeutic purposes. Development of ASPH-specific antibodies has been described in several articles [101–105]. The human IgG1 PAN-622 recognizes the catalytic domain of ASPH. This antibody is not directly cytotoxic for tumor cells but is internalized and can deliver cytotoxic moieties into cells [84]. In the subsequent study with a mouse model of metastatic breast cancer, PAN-622 was used for bioimaging and radioimmunotherapy with promising results [104]. Mouse IgG1 monoclonal antibody binding to the C-terminal ASPH domain mediated ADCC by human NK cells [103].

Recently, a second-generation antibody approach has been disclosed. The prepared antibody binds to the extreme C-terminus of ASPH (US 20190382506) that is involved in specific substrate recognition [106]. Therefore, this antibody has direct ASPH inhibitory activity and does not require any radioisotope or cytotoxic payload for potential therapeutic activity.

## Conclusions

**Fig. 4:**
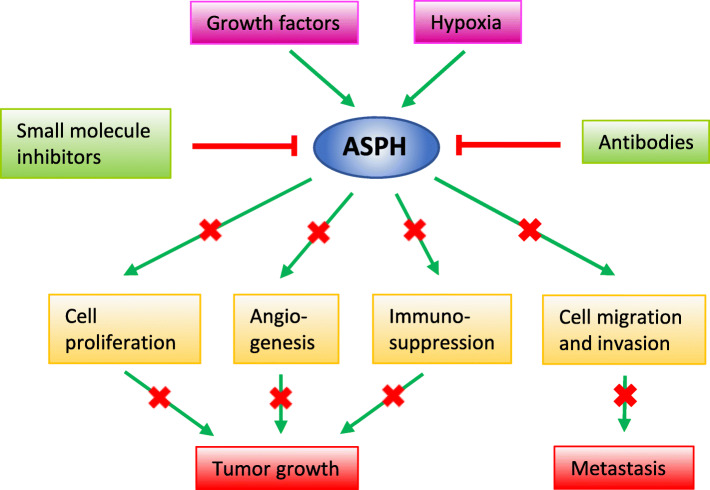
ASPH as a therapeutic target. ASPH expression is upregulated by growth factors and hypoxia. Its enzymatic activity can be inhibited by SMIs or monoclonal antibodies which results in reduction of cell proliferation, angiogenesis, immunosuppression, and cell migration and invasion. Consequently, tumor growth and metastasis are also reduced

ASPH is an important enzyme in malignant transformation of cells. It stimulates tumor cell proliferation, migration and invasion but it can also affect other cells in tumor microenvironment. Two main pathways, Notch and SRC, through which ASPH promotes the tumor growth have been identified. It has also been shown that ASPH expression is induced by some growth factors and hypoxia and is regulated at various levels. The overexpression of ASPH and its downstream targets has been detected in numerous human malignancies. Since ASPH is not expressed in appreciable level in normal adult tissues and the catalytic domain is localized on the cell surface, it has been proposed as one of the most exciting potential therapeutic targets (Fig. 4). Small inhibitory molecules, orally bioavailable, have been developed and successfully tested in several cancer models but they have not yet advanced into clinical trials. Additionally, as ASPH was identified as a tumor-associated antigen, immunotherapy approaches, vaccines and monoclonal antibodies, were tested with promising results in preclinical experiments and results of phase I clinical trial with the PAN-301-1 vaccine were published [100].

Despite the progress in understanding ASPH involvement in signaling pathways, the high number of other potential targets of ASPH hydroxylation suggests that the ASPH impact on tumor biology might be more complex, including potential impact on the transforming growth factor beta (TGF-β), growth arrest specific 6/AXL receptor tyrosine kinase (Gas6/Axl), and Wnt pathways. Even a potential effect of ASPH and its inhibitors on immune cells through the well identified ASPH target, Notch signaling, has not been sufficiently investigated, yet, and should be examined in future studies.

## Supplementary information

**Additional file 1: Figure S1.** Experimental and computationally predicted ASPH substrates.

## Data Availability

Not applicable.

## Abbreviations

2OG: 2-oxoglutarate
ADAM: A disintegrin and metallopeptidase domain
ADCC: Antibody-dependent cellular cytotoxicity
ADCP: Antibody-dependent cellular phagocytosis
AMPK: AMP-activated protein kinase
ASPH: Aspartate β-hydroxylase
AXL: AXL receptor tyrosine kinase
cbEGF: Calcium-binding epidermal growth factor
CCND1: Cyclin D1
CD: Cluster of differentiation
CDC: Complement dependent cytotoxicity
CDK: Cyclin-dependent kinase
CK2: Casein kinase 2
DLL: Delta like
CSL: *C*BF-1/RBP-jκ, *S*u(H), *L*ag-1
EGF: Epidermal growth factor
EMT: Epithelial-to-mesenchymal transition
EPCAM: Epithelial cell adhesion molecule
ERK: Extracellular signal-regulated kinase
FIH: Factor inhibiting HIF
FTO: Fat mass and obesity protein
GAS6: Growth arrest specific 6
GSK-3β: Glycogen synthase kinase-3β
HCC: Hepatocellular carcinoma
HLA: Human leukocyte antigen
HES1: Hes family bHLH transcription factor 1
HEY1: HES with YRPW motif 1
HIF-1α: Hypoxia inducible factor 1 alpha
IGF: Insulin-like growth factor
IHC: Immunohistochemistry
IN: Insulin
IRS1: Insulin receptor substrate 1
JAG: Jagged
KDM4E: Lysine-specific demethylase 4E
MAPK: Mitogen-activated protein kinase
MEF2: Myocyte enhancer factor 2
MMP: Metallopeptidase
MYC: MYC proto-oncogene
NICD: Notch intracellular domain
NFκB: Nuclear factor kappa B
NK: Natural killer
PCNA: Proliferating cell nuclear antigen
PDGF: Platelet derived growth factor
PHD: Prolyl hydroxylase domain
PI3K/Akt: Phosphatidylinositol-3-kinase/protein kinase B
PKA: Protein kinase A
PKC: Protein kinase C
pRB: Retinoblastoma protein
PTGS2: Prostaglandin-endoperoxide synthase 2 (cyclooxygenase 2)
RT-qPCR: Reverse transcription quantitative polymerase chain reaction
SERCA: Sarco/endoplasmic reticulum calcium ATPase
SRC: SRC tyrosine kinase
SMI: Small molecule inhibitor
TAA: Tumor associated antigen
TCF/LEF: T-cell factor/lymphoid enhancer-binding factor
TGF-β: Transforming growth factor beta
TNFA: Tumor necrosis factor alpha
VEGF: Vascular endothelial growth factor
