# Supplementary material for: Aspartate β-hydroxylase as a target for cancer therapy
Source: J Exp Clin Cancer Res. 2020 Aug 18;39:163. doi: 10.1186/s13046-020-01669-w (PMC7433162; doi:10.1186/s13046-020-01669-w)
Supplement: Supplementary file 1 — Additional file 1: Figure S1. Experimental and computationally predicted ASPH substrates. [file 13046_2020_1669_MOESM1_ESM.pdf]

Supplementary Figure 1: Experimental and computationally predicted ASPH substrates. Panel A, Notch signaling proteins; panel B, lipid receptor proteins; panel C, blood coagulation proteins; panel D, thrombospondins; panel E, innate immune system proteins; panel F, fat proteins; panel G, bone related proteins; panel H, seven transmembrane domain containing proteins; panel I, fibrillins and TGF-beta containing proteins; panel J, platelet and EGF proteins; panel K, retina and eye-related proteins; panel L, metastatic breast cancer proteins; panel M, SLIT proteins; panel N, miscellaneous proteins.

Abbreviations:
NOTCH1: notch receptor 1; NOTCH2: notch receptor 2; NOTCH3: notch receptor 3; NOTCH4: notch receptor 4; JAG1: jagged canonical Notch ligand 1; JAG2: jagged canonical Notch ligand 2; DLL1: delta like canonical Notch ligand 1; DLL3: delta like canonical Notch ligand 3; DLL4: delta like canonical Notch ligand 4; DNER: delta/notch like EGF repeat containing; DLK1: delta like non-canonical Notch ligand 1; DLK2: delta like non-canonical Notch ligand 2; CRB1: crumbs cell polarity complex component 1; CRB2: crumbs cell polarity complex component 2; LDLR: low density lipoprotein receptor; VLDLR: very low density lipoprotein receptor; LRP1: LDL receptor related protein 1; LRP1B: LDL receptor related protein 1B; LRP2: LDL receptor related protein 2; LRP4: LDL receptor related protein 4; LRP8: LDL receptor related protein 8; FVII: coagulation factor VII; FIX: coagulation factor IX; FX: coagulation factor X; TM: thrombomodulin; PROC: protein C, inactivator of coagulation factors Va and VIIIa; PROZ: protein Z, vitamin K dependent plasma glycoprotein; PROS: protein S; GAS6: growth arrest specific 6; THBS1: thrombospondin 1; THBS2: thrombospondin 2; THBS3: thrombospondin 3; THBS4: thrombospondin 4; CD93: CD93 molecule; MASP1: mannan binding lectin serine peptidase 1; MASP2: mannan binding lectin serine peptidase 2; C1R: complement C1r; C1S: complement C1s; FAT1: FAT atypical cadherin 1; FAT2: FAT atypical cadherin 2; FAT3: FAT atypical cadherin 3; FAT4: FAT atypical cadherin 4; COMP: cartilage oligomeric matrix protein; BMP1: bone morphogenetic protein 1; CELSR1: cadherin EGF LAG seven-pass G-type receptor 1; CELSR2: cadherin EGF LAG seven-pass G-type receptor 2; CELSR3: cadherin EGF LAG seven-pass G-type receptor 3; ADGRE5: adhesion G protein-coupled receptor E5; ADGRL4: adhesion G protein-coupled receptor L4; ADGRE1: adhesion G protein-coupled receptor E1; ADGRE2: adhesion G protein-coupled receptor E2; ADGRE3: adhesion G protein-coupled receptor E3; ADGRE4: adhesion G protein-coupled receptor E4, pseudogene; FBN1: fibrillin 1; FBN2: fibrillin 2; FBN3: fibrillin 3; LTBP1: latent transforming growth factor beta binding protein 1; LTBP2: latent transforming growth factor beta binding protein 2; LTBP3: latent transforming growth factor beta binding protein 3; LTBP4: latent transforming growth factor beta binding protein 4; SCUBE1: signal peptide, CUB domain and EGF like domain containing 1; SCUBE2: signal peptide, CUB domain and EGF like domain containing 2; SCUBE3: signal peptide, CUB domain and EGF like domain containing 3; MMRN1: multimerin 1; EGF: epidermal growth factor; HMCN1: hemicentin 1; HMCN2: hemicentin 2; EYS: eyes shut homolog; SNED1: sushi, nidogen and EGF like domains 1; SVEP1: sushi, von Willebrand factor type A, EGF and pentraxin domain containing 1; SLIT1: slit guidance ligand 1; SLIT2: slit guidance ligand 2; SLIT3: slit guidance ligand 3; FBLN1: fibulin 1; FBLN2: fibulin 2; EFEMP1: EGF containing fibulin extracellular matrix protein 1; EFEMP2: EGF containing fibulin extracellular matrix protein 2; FBLN5: fibulin 5; FBLN7: fibulin 7; VWCE: von Willebrand factor C and EGF domains; MEGF6: multiple EGF like domains 6; AGRN: agrin; NCAN: neurocan; NPNT: nephronectin; EDIL3: EGF like repeats and discoidin domains 3; NELL1: neural EGFL like 1; NELL2: neural EGFL like 2; NRXN1: neurexin 1; HEG1: heart development protein with EGF like domains 1; SUSD1: sushi domain containing 1; CD248: CD248 molecule; TPO: thyroid peroxidase; CRELD1: cysteine rich with EGF like domains 1; MEGF8: multiple EGF like domains 8.

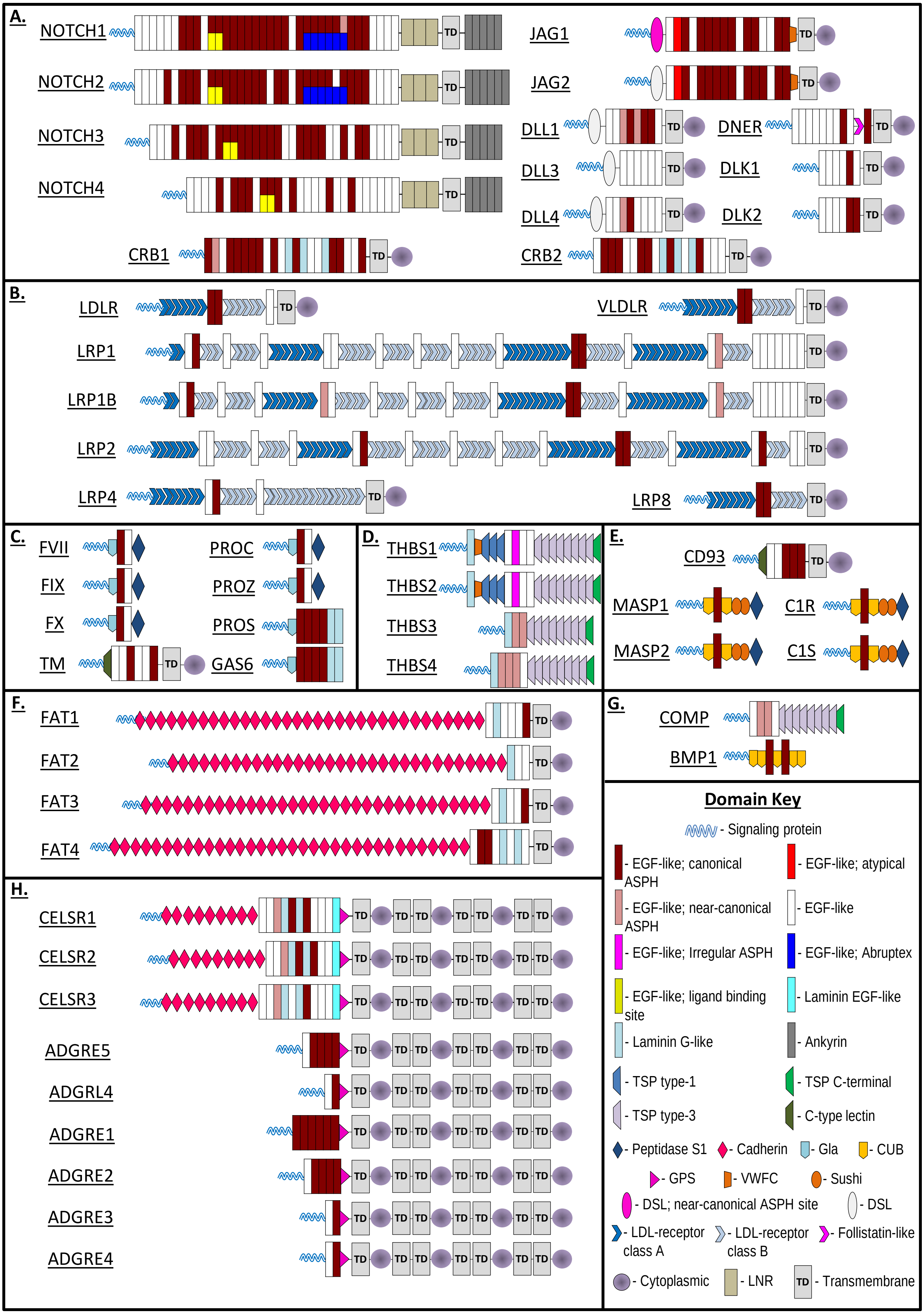

A.
NOTCH1
NOTCH2
NOTCH3
NOTCH4
CRB1
JAG1
JAG2
DLL1
DNER
DLL3
DLK1
DLL4
DLK2
CRB2
B.
LDLR
VLDLR
LRP1
LRP1B
LRP2
LRP4
LRP8
C.
FVII
PROC
FIX
PROZ
FX
PROS
TM
GAS6
D.
THBS1
THBS2
THBS3
THBS4
E.
CD93
MASP1
C1R
MASP2
C1S
F.
FAT1
FAT2
FAT3
FAT4
G.
COMP
BMP1
H.
CELSR1
CELSR2
CELSR3
ADGRE5
ADGRL4
ADGRE1
ADGRE2
ADGRE3
ADGRE4
TD
Domain Key
- Signaling protein
- EGF-like; canonical ASPH
- EGF-like; atypical
- EGF-like; near-canonical ASPH
- EGF-like
- EGF-like; Irregular ASPH
- EGF-like; Abruptex
- EGF-like; ligand binding site
- Laminin EGF-like
- Laminin G-like
- Ankyrin
- TSP type-1
- TSP C-terminal
- TSP type-3
- C-type lectin
- Peptidase S1
- Cadherin
- Gla
- CUB
- GPS
- VWFC
- Sushi
- DSL; near-canonical ASPH site
- DSL
- LDL-receptor class A
- LDL-receptor class B
- Follistatin-like
- Cytoplasmic
- LNR
- Transmembrane

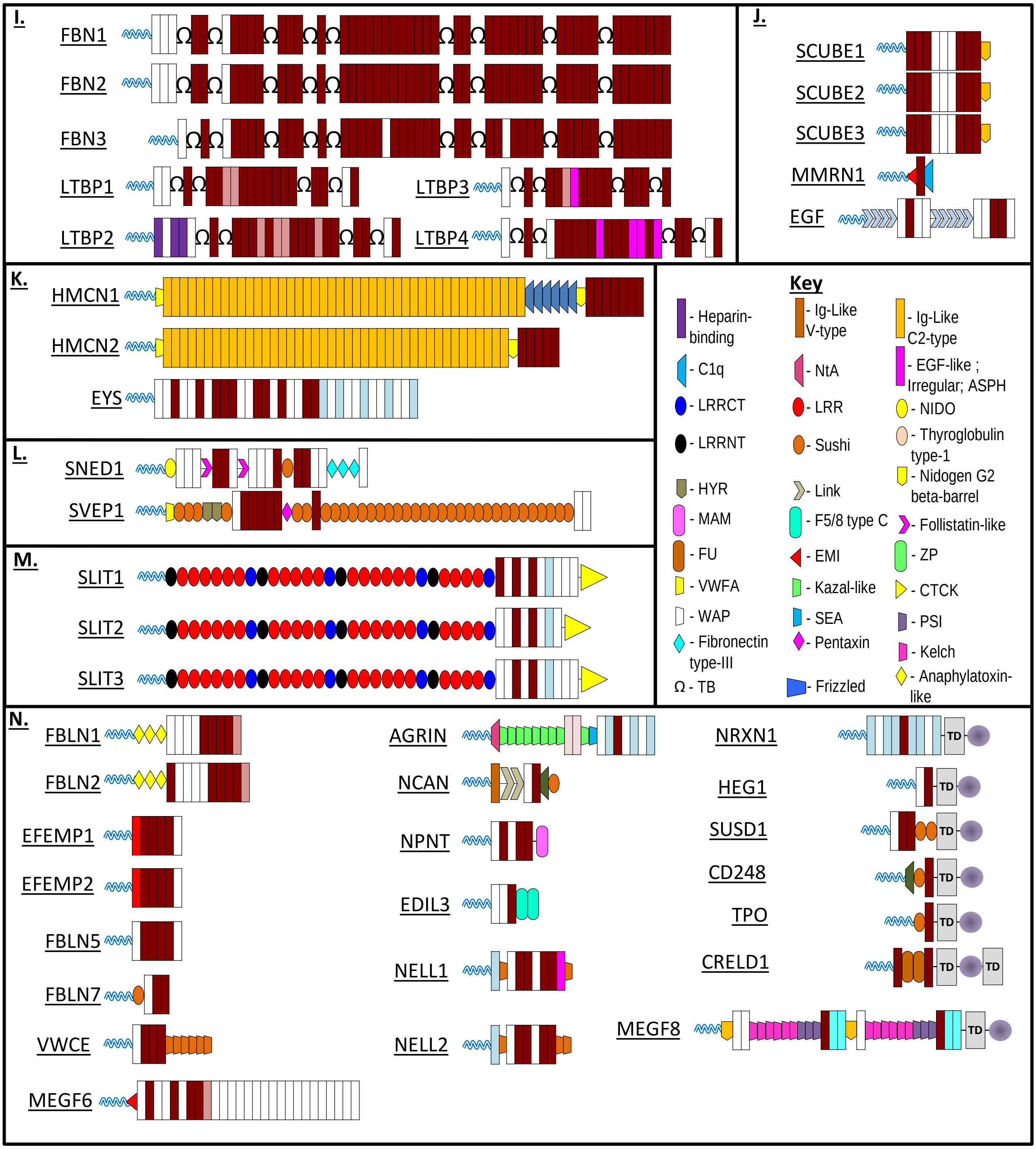
I.
FBN1
FBN2
FBN3
LTBP1
LTBP3
LTBP2
LTBP4
J.
SCUBE1
SCUBE2
SCUBE3
MMRN1
EGF
K.
HMCN1
HMCN2
EYS
L.
SNED1
SVEP1
M.
SLIT1
SLIT2
SLIT3
Key
- Heparin-binding
- Ig-Like V-type
- Ig-Like C2-type
- C1q
- NtA
- EGF-like ; Irregular; ASPH
- LRRCT
- LRR
- NIDO
- LRRNT
- Sushi
- Thyroglobulin type-1
- HYR
- Link
- Nidogen G2 beta-barrel
- MAM
- F5/8 type C
- Follistatin-like
- FU
- EMI
- ZP
- VWFA
- Kazal-like
- CTCK
- WAP
- SEA
- PSI
- Fibronectin type-III
- Pentaxin
- Kelch
Ω - TB
- Frizzled
- Anaphylatoxin-like
N.
FBLN1
FBLN2
EFEMP1
EFEMP2
FBLN5
FBLN7
VWCE
MEGF6
AGRIN
NCAN
NPNT
EDIL3
NELL1
NELL2
NRXN1
HEG1
SUSD1
CD248
TPO
CRELD1
MEGF8
TD
